# A Biomimetic Approach to Diode Laser Use in Endodontic Treatment of Immature Teeth: Thermal, Structural, and Biological Analysis

**DOI:** 10.3390/biomimetics10040216

**Published:** 2025-04-02

**Authors:** Dijana D. Mitic, Maja S. Milosevic Markovic, Igor D. Jovanovic, Dragan D. Mancic, Kaan Orhan, Vukoman R. Jokanovic, Dejan Lj. Markovic

**Affiliations:** 1 School of Dental Medicine, University of Belgrade, 11000 Belgrade, Serbia; maja.milosevic@stomf.bg.ac.rs (M.S.M.M.); dejan.markovic@stomf.bg.ac.rs (D.L.M.); 2 Faculty of Electronic Engineering, University of Nis, 18000 Nis, Serbia; igor.jovanovic@elfak.ni.ac.rs (I.D.J.); dragan.mancic@elfak.ni.ac.rs (D.D.M.); 3 Department of Dentomaxillofacial Radiology, Faculty of Dentistry, Ankara University, 06000 Ankara, Turkey; call53@yahoo.com; 4 Vinca Institute of Nuclear Sciences, University of Belgrade, 11000 Belgrade, Serbia; vukoman@vinca.rs

**Keywords:** 940 nm diode laser, regenerative endodontic treatment, immature permanent teeth, SCAPs, micro-CT, thermographic measurement

## Abstract

The root walls of immature permanent teeth are often weak, thin, and short, making regenerative endodontic treatment (RET) necessary. The goal of RET is to create a favorable environment for further root development. A biomimetic approach is essential for thorough disinfection, followed by the preservation and potential stimulation of stem cells from surrounding tissue to enable root regeneration and continued development. The objective of this study was to assess temperature changes on the external root surface, structural alterations in the internal root walls following irradiation with a 940 nm diode laser, and the biocompatibility of stem cells from the apical papilla (SCAPs). Irradiation was performed with varying output powers (0.5 W, 1 W, 1.5 W, and 2 W) in continuous mode for 5 s over four consecutive cycles. Thermographic measurements during irradiation, the micro-CT analysis of root samples, and mitochondrial activity of SCAPs were evaluated. The heating effect correlated directly with a higher output power and thinner root walls. A 1 W output power was found to be safe for immature teeth, particularly in the apical third of the root, while 1.5 W could be safely used for mature mandibular incisors. Diode laser irradiation at 1 W and 1.5 W significantly stimulated SCAPs’ mitochondrial activity within 24 h post-irradiation, indicating a potential photobiostimulatory effect. However, no significant changes were observed at lower (0.5 W) and higher (2 W) output powers. The area of open tubular space inside the root canal was significantly reduced after irradiation, regardless of the applied power. Additionally, irradiation contributed to the demineralization of the dentin on the inner root walls. Future studies should explore the impact of irrigants used between irradiation cycles, the potential benefits of conical laser tips for more even energy distribution, and a thorough analysis of how disinfection protocols affect both the dentin structure and stem cell viability.

## 1. Introduction

The primary cause of endodontic pathology is infection, which occurs when microorganisms invade the pulp chamber and the complex root canal system. These microorganisms form biofilms and establish reservoirs within the dentinal tubules, where they are protected from the immune system, systemic antibiotics, and even irrigating solutions [1]. A microbial presence in persistent root canal infections, despite chemo-mechanical preparation and intracanal medications, remains a major cause of treatment failure before obturation [2]. Conventional chemical substances used for intracanal disinfection have limited potential for intratubular penetration [3], while bacterial invasion occurs about ten times deeper into dentin [4], depending on the irrigation solution [5], and the recolonization of root canal space often occurs after treatment. Endodontic treatment for necrotic immature permanent teeth presents even more significant challenges. Chemo-mechanical debridement is less effective in immature teeth than in mature ones [6], as the amount of instrumentation and the use of aggressive irrigants are often limited.

The root walls of immature permanent teeth are often weak, thin, and short, making regenerative endodontic treatment (RET) necessary. The goal of RET is to create a favorable environment for continued root development, root elongation, and an increase in the dentin volume. A biomimetic approach is necessary in regenerative procedures of the inflamed pulp of immature teeth as providing thorough disinfection [7] is important for the preservation and possible stimulation of stem cells from the surrounding tissue that are able to regenerate root tissue and manage further development. The use of lasers could support the body’s natural healing mechanisms during regenerative endodontics. Various laser systems have been suggested to improve disinfection procedures in endodontics [8], among them, near-infrared diode lasers with the main advantage of deep dentin disinfection [9]. A key advantage of using lasers in endodontics, as opposed to traditional disinfecting agents [10], is their ability to effectively reduce bacteria without causing subsequent harsh effects. This mimics the body’s natural immune response, where pathogens are eliminated through a precise, targeted action, allowing for a more natural recovery. On the other hand, diode lasers could stimulate collagen production, modulate inflammation and oxidative stress levels [11], and improve periapical healing [12], in that way promoting natural tissue regeneration. A focused laser beam is a minimally invasive tool that enables regeneration while minimizing trauma to surrounding tissues.

When the root dentin is exposed to diode laser irradiation, the light energy is converted into heat, providing effective additional root canal disinfection, particularly against *Enterococcus faecalis* [13], and when used in combination with chlorhexidine [14]. Typically operating in the 810–980 nm range, the 940 nm diode laser is particularly effective for eliminating bacteria and removing the smear layer, while ensuring that thermal levels remain safe [15]. Additionally, the near-infrared laser has high transmission through water and strong absorption by melanin and hemoglobin. Due to its low absorption in water, the laser energy can penetrate deeply into dentin, raising the bacterial cell temperature and inducing cell death [16]. This effect is primarily due to melanin in bacterial membranes, which makes them highly sensitive to temperature changes, ultimately resulting in bacterial cell death. Due to the high water content in root canal dentin, these lasers achieve deep penetration and effective disinfection [17]. On the other hand, the rise in temperature in the root canal could subsequently affect the dentin ultrastructure on inner root walls, causing the crystallization of inorganic dentin tissue [18]. High-resolution X-ray micro-computed tomography (micro-CT) as an advanced, non-invasive 3D imaging method could be used for the in situ analysis of possible temperature effects on the inner root canal walls [19].

Laser energy applied to the intracanal dentin can lead to temperature increases that exceed biologically safe limits at the external root surface, as well as periodontal and periapical tissues, as the energy transfers through the tissue. Depending on the chosen laser protocol for endodontic disinfection, different root segments could be more prone to photothermal injury [20]. The apical root segment is considered even more sensitive considering the apical papilla tissue nearby, consistent of stem cells from apical papilla (SCAPs), involved in the formation of the apical root segment [21]. The remaining dentin thickness may influence the photothermal effects, which could also impact cells in the apical papilla. However, additional research is required to gain a deeper understanding of these effects.

The application of diode lasers in RET could present a new treatment approach, but safety must be carefully evaluated. Before clinical studies involving diode lasers used for RET on immature permanent teeth, a standardized protocol should be developed. A temperature variation of 10 °C on the root surface in in vivo studies is generally considered safe for periodontal tissue due to its high vascularization [22]. Since temperature decreases during in vitro conditions occur more rapidly, 7 °C has been suggested as a biologically accepted limit on the surface of extracted teeth [23], and it was used in the present study as a threshold level. While diode lasers have been explored in the context of endodontic treatment, a significant gap remains in the literature regarding their specific application in regenerative endodontics, particularly in immature permanent teeth. The comprehensive evaluation of external root surface heating on both mature and immature permanent teeth, structural changes within the root canal walls, and the viability of SCAPs under various laser power settings would provide valuable new insights into optimizing diode laser protocols for safer and more effective regenerative endodontic procedures.

The objective of this study was to assess, in vitro, temperature changes on the mesial external root surface of mature and immature permanent mandibular human incisors, structural alterations in the internal root walls following irradiations, and the viability of SCAPs during endodontic laser treatment, when grading output powers of a 940 nm diode laser were applied.

## 2. Materials and Methods

This study was performed at the School of Dental Medicine, University of Belgrade, approved by the Ethics Committee (no. 36/8), and carried out in compliance with the Declaration of Helsinki.

### 2.1. Diode Laser System

The commercial, Class 4 diode laser system in the mode for additional disinfection during endodontic treatment was used. The 940 ± 10 nm wavelength Epic laser (Biolase, San Clemente, CA, USA), with 10 W maximal power and plain-ended flexible endodontic fiber (200 µm diameter). The laser was calibrated prior to use, by manufacturers. Following the manufacturer’s recommendations, 940 nm laser fiber was changed between measuring groups, and non-initiated fibers were used.

### 2.2. Sample Size

The sample size was calculated as suggested previously [24]. The calculation was based on the mean difference during irradiation with the lowest applied output power (0.5 W) in mature and immature root models, with two-sided α set on 0.05, β = 0.2, and 80% power of this study. It was calculated on the expected smallest difference in temperature variations. The sample size was calculated on 5 samples per group.

Thus, forty-six incisors were included in this study, twenty samples for mature root preparation and twenty samples for immature root preparation, both divided in four groups, based on irradiation output power used. Six remaining samples were used for additional structural study, two samples per group.

### 2.3. Samples’ Preparation

Intact mandibular human incisors, extracted for periodontal reasons, were collected with patients’ informed written consents. All samples were X-rayed to verify the morphology of a single root with a single canal. Teeth were stored in sterile phosphate-buffer saline (PBS; Sigma-Aldrich, St. Louis, MO, USA), at +4 °C, for no longer than 15 days prior to the experiment.

#### 2.3.1. Preparation of Mature Root Samples

**Figure 1:**
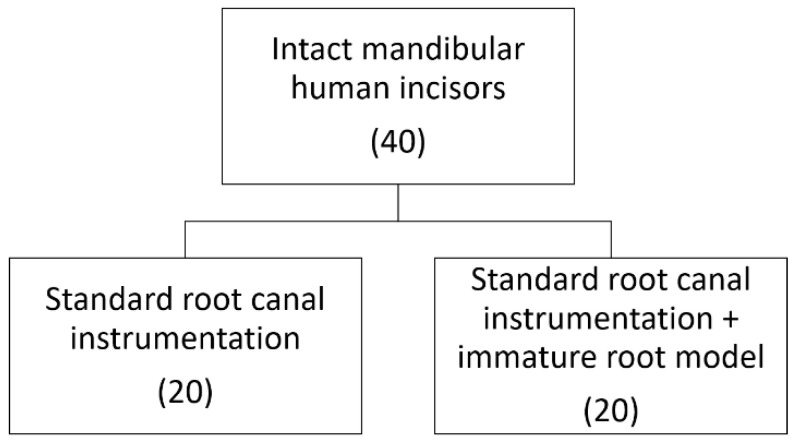
Flowchart of samples’ preparation.

For the irradiation of mature permanent root samples, root canals were instrumented to the apical size of #40 with the step-back instrumentation technique by using K-type files and rinsed with 2% sodium hypochlorite (Chloraxid, Cerkamed, Stalowa Wola, Poland) in between each file. The working length was set 1 mm shorter than the apical foramen (at 9 mm root length). At the end of instrumentation, canals were rinsed with 18% EDTA (Ultradent, Cologne, Germany) for 1 min, followed by 2% sodium hypochlorite and saline irrigation, dried with paper points, and subjected to laser treatments (Figure 1).

#### 2.3.2. Preparation of Immature Root Samples

After standard endodontic preparation, samples were further instrumented under constant sterile saline irrigation to the parallel-wall canal 1.3 mm in diameter, by using an 130 Peeso reamer (FKG Dentaire SA, La Chaux-de-Fonds, Switzerland) to create immature permanent teeth model as previously described [25]. The working length was also set 1 mm shorter than the apical foramen. At the end of instrumentation, canals were rinsed with 18% EDTA for 1 min, followed by 2% sodium hypochlorite and saline irrigation, dried with paper points, and subjected to laser treatments (Figure 1). Digital radiographs of all samples were made by the digital radiography (Trophy Radiologie, Croissy-Beaubourg, France) and the thickness of the sample’s mesial root canal wall was measured.

### 2.4. Simulation of Endodontic Laser Treatment

Prepared mature root samples were dried with paper points, randomly distributed to four main groups, based on used output power, and set on plastic holders. Laser irradiations were applied in dry canals to avoid any fluid agitation effects, potential dentin ablation, or additional laser absorption into water. The fiber was positioned at the working length, and the laser was activated while being moved in a helicoidal motion from the apical to the coronal end (1–2 mm/s) for 5 s, four consecutive times, with 20 s resting periods in between [23,26]. Irradiations were performed with grading output powers (0.5 W, 1 W, 1.5 W, and 2 W) in continuous mode, which corresponds to total radiant energy of 10 J/cm^2^, 20 J/cm^2^, 30 J/cm^2^, and 40 J/cm^2^, respectively. Detail irradiation parameters and calculations are given in Table A1.

In the second part of this study, immature root samples were randomly distributed to four main groups depending on output power, as described for mature root samples. Roots were dried with paper points and set on plastic holders, and the same irradiation treatments were applied as described for mature root samples so compared results would be obtained.

### 2.5. Temperature Measurements

The samples were placed in plastic holders in a standardized vertical orientation, with the mesial root surface directed toward the thermographic camera (Varioscan^®^ high-resolution model 3021, Jenoptik, Dresden, Germany). The camera with 0.03 °C thermal resolution and 8–12 µm of spectral range was positioned at 0.5 m from the samples. Factory-calibrated absolute accuracy of the measurements, at room temperature of 22 ± 2 °C, was less than ±2 K. The temperature was measured during four irradiation cycles and following resting periods. Peak temperature values from all root thirds were recorded (Figure S1). The camera’s lens system captured infrared radiation emitted from the root surface, while the photosensor converted this energy into electrical impulses. The temperature values were then transformed into images displayed on the screen. The obtained thermograms were analyzed using IRBIS Professional 2.2 software (InfraTec GmbH, Dresden, Germany). Measurements were performed in a controlled ambient temperature at 23 ± 2 °C. Furthermore, the temperature on the inner dentinal walls was calculated based on the average values of temperature measurements from the external root surface, along with the thermal conductivity of the root cement and dentin, and the root’s topology. A detailed explanation and results are given in Appendix A.

### 2.6. Micro-CT Scanning

The samples were scanned using a high-resolution desktop micro-CT system (Skyscan 1275, Skyscan, Kontich, Belgium). As detailed in Section 2.3.1, the samples were instrumented and treated with a diode laser (1 W and 2 W), as described in Section 2.4. Non-irradiated samples served as controls. Each sample was rinsed and stored in a saline solution. The samples were positioned upright on the platform for scanning, and the roots were secured with wax. Scanning was performed at 100 kVp, with a 0.5 mm Al filter, 100 mA beam current, 4.73 μm pixel size, 0.5° rotation steps, and three-frame averaging.

Additionally, to reduce ring artifacts, detector air calibration was conducted in between the samples. The parameters were optimized for high-resolution imaging while reducing beam hardening artifacts and noise, ensuring accurate visualization of structural changes with a reasonable scanning time. A correction of beam hardening (40%) and ring artifact (0) were applied. Full circle rotation for every sample was carried out over a 5 min integration period, with the average scanning time for each sample being approximately 4 h.

#### 2.6.1. Micro-CT Image Reconstructions

**Figure 2:**
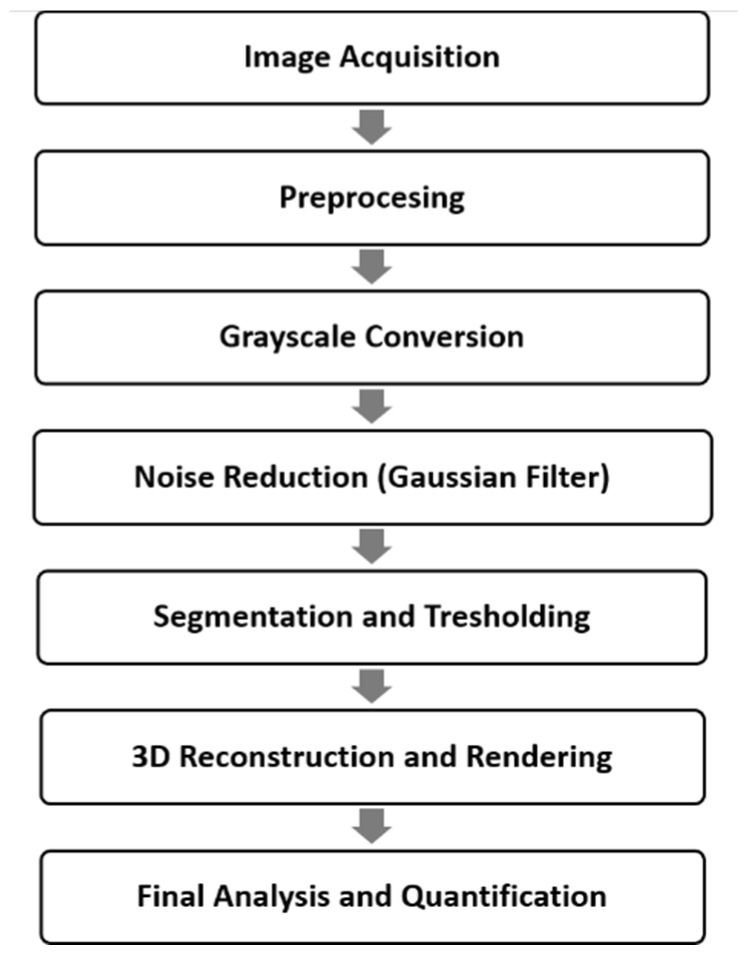
Flowchart of micro-CT analysis.

NRecon software (v 1.7.1.0, Skyscan, Kontich, Belgium) was used for reconstructions with algorithm modification described by Feldkamp et al. [27]. This algorithm utilizes a three-dimensional density function derived from a series of two-dimensional projections. The NRecon software, incorporating this algorithm, was used to create axial two-dimensional images. Prior to reconstruction, settings such as beam-hardening correction, as previously described, and optimal contrast limits (0–0.0005) were applied. These contrast limits followed SkyScan’s guidelines, where the minimum limit was set to zero to ensure the density scale began at zero, and the maximum limit corresponded to the highest brightness value, representing the highest density. The image dataset consisted of approximately 1800 axial tomographic slices, each measuring 1024 × 1024 pixels with a 16-bit gray level (Figure S2). All reconstructions and measurements were conducted on a 21.3-inch flat-panel color-active matrix TFT medical display (NEC MultiSync MD215MG, Muenchen, Germany) with a resolution of 2048 × 2560 at 75 Hz, a 0.17 mm dot pitch, and operated at 11.9 bits (Figure 2).

#### 2.6.2. Volumetric Rendering Software Analysis

After obtaining the axial images from Micro-CT scanning, the original grayscale images were processed using a Gaussian low-pass filter for noise reduction. An automatic segmentation threshold was then applied to separate the teeth from the pulp. The original color images were converted to grayscale using CTAn (ver. 1.16.1.0, SkyScan, Kontich, Belgium). The Gaussian low-pass filter helped eliminate noise and improve contrast. The images were subsequently rendered into volumetric images and reconstructed sagittal, axial, and coronal slices, along with 3D models, were generated (Figure S2). By making the cement and dentin translucent, the spatial relationship between the root contour and the pulp was visualized in three dimensions.

#### 2.6.3. Measurements

The morphometric relationship between the tooth and pulp was measured using CTAn software (ver. 1.16.1.0, SkyScan, Kontich, Belgium). Using the software, the total tooth volume, total pulp volume, and the tooth/pulp ratio were also calculated. CTAn allows users to “sculpt out” the desired volume from 3D structure by adjusting opacity and brightness, so the unwanted voxels are removed prior to final tooth and pulp volumes being calculated. Entire specimens’ volume is used for measurement of six parameters: area of open tubular space (pixel^2^ or µm^2^), area of closed tubular space (pixel^2^ or µm^2^), closed porosity (percent), open porosity (percent), total area of tubular space (pixel^2^ or µm^2^), and the zone of demineralization. These measurements were taken from the root. An open pore was defined as one that intersected the boundary of the region of interest (ROI), indicating it was connected to the external environment in either 2D or 3D. In contrast, a closed pore was not connected to the outside in either dimension. Closed pores appeared as black pixels surrounded by white pixels, representing dentin tubules that remained unsealed. The total tubular space area was calculated as the combined area of all pores (both open and closed) as a percentage of the total ROI, which in this case, was within a 3 × 3 mm region. The demineralization zone was defined by measuring the perpendicular distance from the pulp periphery to the furthest point of the visualized density difference within the root (Figure S2).

### 2.7. In Vitro Biocompatibility on Stem Cells from Apical Papilla (SCAPs)

#### 2.7.1. Isolation and Characterization

Cells from apical papilla tissue were isolated as previously described [28]. Tissues from the apical papilla were obtained from third molars of healthy patients (aged 18–21 years) who required tooth extraction for orthodontic reasons. The tissues were immediately transferred to complete medium consisting of Gibco Dulbecco’s modified Eagle’s F12 (DMEM/F12), supplemented with 10% fetal bovine serum (FBS) and 1% antibiotic/antimycotic (ABAM) solution, all from Thermo Fisher Scientific, Inc., Waltham, MA, USA. Within one hour of extraction, the tissues were rinsed in PBS, minced, and processed using the outgrowth isolation method to obtain stem cells from the apical papilla (SCAPs), as described previously [29]. The tissues were cultured in complete medium and regularly passaged. At passage four, 1 × 10^6^/mL of cells was used for labeling with each of selected conjugated monoclonal antibodies for mesenchymal markers—CD73 (PB) (Sony Biotechnology, San Jose, CA, USA), CD90 (FITC) (Life Technologies, Carlsbad, CA, USA), and CD105 (FITC) (Exbio, Prague, Czech Republic)—and hematopoietic markers—CD34 (FITC) (Sony Biotechnology, San Jose, CA, USA) and CD45 (PE) (Exbio, Prague, Czech Republic). Antibodies’ incubation for 45 min was followed by PBS rinsing and 2% formaldehyde fixation. Samples were stored at 4 °C overnight. The following day, cells were analyzed using a flow cytometer CyFlow® Space Partec (Partec GmbH, Münster, Germany). Cells from the fifth passage were used for this study.

#### 2.7.2. Mitochondrial Activity Assay (MTT)

For MTT assay, cells were seeded in every other well of 96-well plate at a concentration of 5 × 10^3^ cells/well. The next day, the medium was changed to 2% FBS medium as it was reported that it is necessary to induce a stress state in the cells just before the treatment when studying photobiomodulation [30]. The next day, medium was discarded, cells were washed with PBS, and freshly added PBS (100 µL/well) was added so that phenol red from the medium did not influence irradiation. Dispersion of laser wave was between 8 and 22°, so by calculating the distance between the distal end of the laser pip and cell monolayer, it was concluded that no dispersion outside well occurs. Cells were irradiated over the transparent plate lid to avoid contamination, over the center of the well, with the laser tip set perpendicular to the plate surface, for 5 s, four consecutive times, with 20 s resting periods in between [26,29]. Irradiations were performed with grading output powers of 0.5 W, 1 W, 1.5 W, and 2 W, in continuous mode, in the dark, under the laminar flow hood. Irradiation parameters are given in Table A1. Following irradiation, PBS from wells was discarded and freshly prepared medium was added to all wells. Plates were incubated at 37 °C in a humidified 5% CO_2_ atmosphere. After 24 h of culturing the medium was discarded, and fresh medium containing 3-(4,5-dimethylthiazol-2-yl)-2,5-diphenyltetrazolium bromide (MTT, 0.5 mg/mL; Sigma-Aldrich) was added. The cells were then incubated as previously described by Castiglioni et al. [31]. After 4 h, the supernatant was discarded, and 100 μL of dimethylsulfoxide (DMSO, Sigma-Aldrich) was added to each well. The plate was then placed on a shaker at 250 rpm for 20 min, in the dark, at 37 °C. Optical density was measured on a microplate reader (RT-2100c, Rayto, Shenzhen, China), at 550 nm. The percentage of MTT reduction was determined in comparison to the control group.

### 2.8. Statistical Analyses

Kolmogorov–Smirnov test was used for normality of data distribution analysis. Differences in temperature variations and samples’ dentin thickness were analyzed by two-way ANOVA followed by Tukey’s post hoc test. Linear regression analysis was performed to estimate if dentin thickness affects temperature variations in mature and immature root samples. For micro-CT measurements, the Kruskal–Wallis test was used. Data were analyzed by Prism software version 9 (GraphPad Software, San Diego, CA, USA). The statistical significance was tested at *p* < 0.05.

## 3. Results

Based on digital radiographic measurements of the dentin thickness in the root thirds of the mesial wall, the overall thickness of the immature root samples was significantly lower compared to the mature samples. Among the mature root samples, no significant differences were observed, as well as among the immature samples (Table 1), and homogenous samples were further allocated to the four main groups depending on their output power.

### 3.1. Temperature Measurements

The peak temperature variation (ΔT) values obtained at the end of the laser irradiation cycles in all three root thirds are presented in Table 2. The heating effect was more evident in immature roots, in all root segments.

The calculated temperatures on the inner root walls, derived from outer surface temperature measurements, the thermal conductivity of both the root cement and dentin, and the root’s topology indicated that heat is transferred through the dentin and cement. However, some of the energy is retained within the inner surface (Table 3).

**Figure 3:**
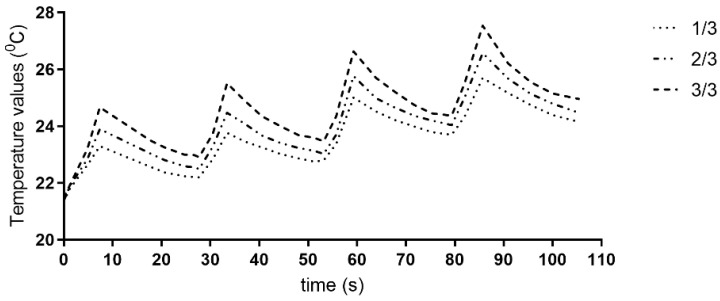
Representative graphs of recorded temperature values in root thirds of the samples for 940 nm diode laser during four irradiation cycles and the following resting periods.

The trend of a rising temperature from the cervical to apical third was observed in all groups, with no statistical significance (*p* > 0.05). The temperature increased as a higher output power was applied. For mature root samples, a power of 2 W raised the ΔT values above 7 °C. Resting periods following irradiation cycles contributed to the temperature decrease (Figure 3, Table S1).

**Figure 4:**
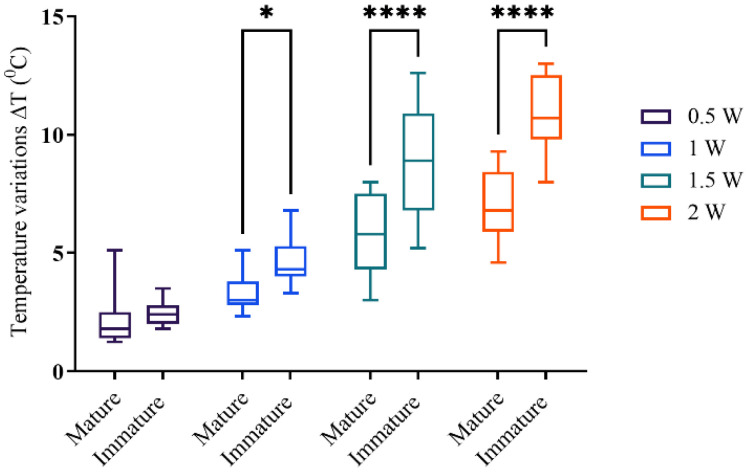
Peak temperature variations at entire external mesial root surfaces at the end of irradiation cycles, in all representative groups. Significant differences between mature and immature roots for the same output power are marked with corresponding symbols, Two-Way ANOVA, * *p* < 0.05, **** *p* ≤ 0.0001.

Immature root samples were more prone to heating than mature root samples when the same output power was applied (Figure 4).

**Figure 5:**
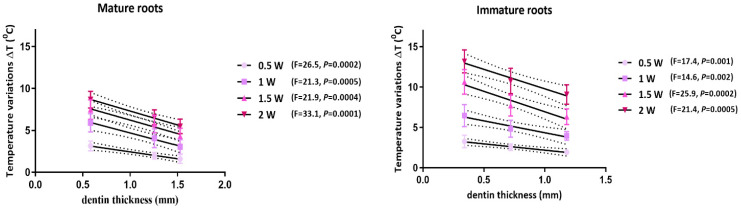
Linear regression analysis of dentin thickness relation to temperature variations during irradiation at four different output powers for 940 nm laser in mature and immature roots. Doted lines represent error bars.

The mean ΔT values were above 7 °C for 1.5 W and 2 W of applied powers. The linear regression analysis revealed a dependency of temperature values on the dentin thickness and output power in mature and immature root samples (Figure 5).

### 3.2. Micro-CT Scanning

**Figure 6:**
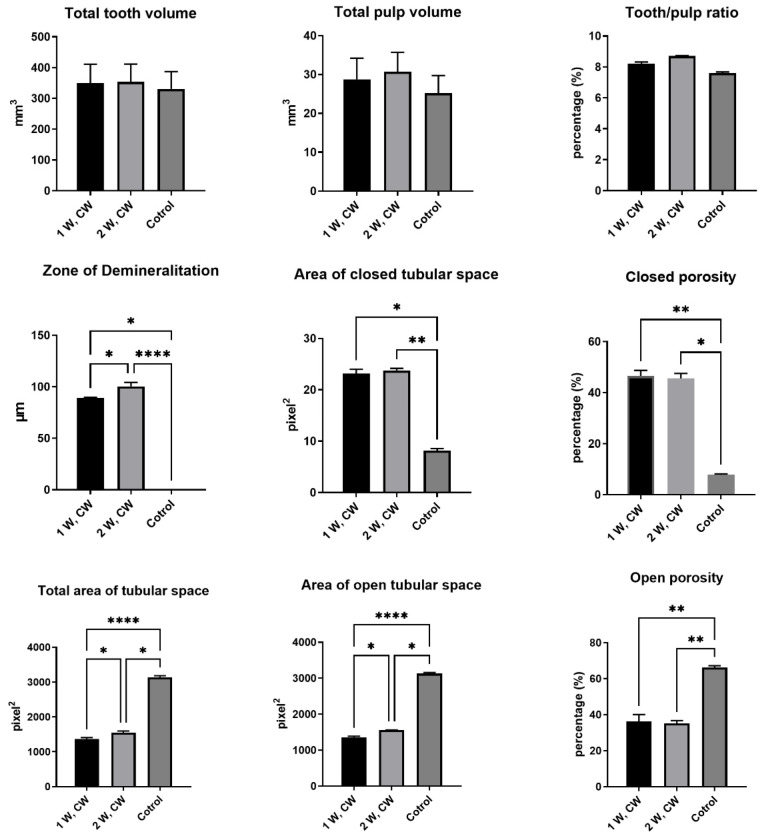
Micro-CT analysis of 940 nm diode laser-irradiated roots, with output powers of 1 and 2 W for 5 s in continuous mode, in 4 cycles, with resting periods (20 s) in between, Kruskal–Wallis test, * *p* < 0.05, ** *p* < 0.01, **** *p* < 0.0001.

Micro-CT analysis of tooth and pulp volume revealed the use of homogenous samples in this study that did not significantly differ in the means of the tooth/pulp ratio. Laser irradiation significantly affected the dentin porosity on the root dentin inner surface regardless of the used output powers (Figure 6). The area of open tubular space inside the root canal was significantly reduced after irradiation, which increased the closed porosity. Also, irradiation with a diode laser contributes to the demineralization of the dentin, i.e., the dentin density on the inner dentin wall.

### 3.3. Flow Cytometry

**Figure 7:**
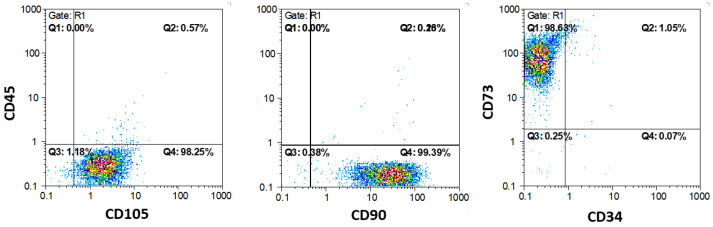
Flow cytometry analysis of CD 45, CD 105, CD 90, CD 73, and CD 34 antibodies on SCAPs at the fifth passage.

The markers of the mesenchymal stem cells (CD 73, CD 90, and CD 105) were positive in 98 to 99% of the cells, while the markers of the hematopoietic stem cells (CD 34 and CD 45) were positive in less than 1% of the cells in the samples (Figure 7). This method confirmed that over 99% of cells from the samples were mesenchymal stem cells.

### 3.4. Mitochondrial Activity Assay (MTT)

**Figure 8:**
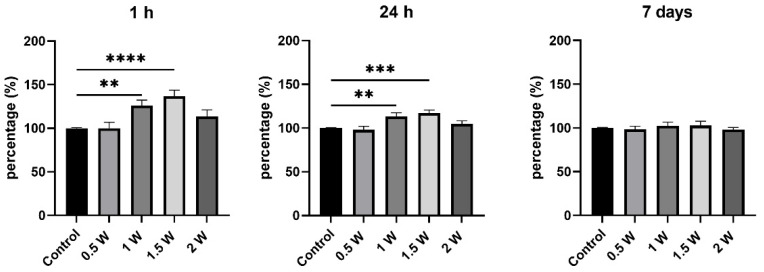
Mitochondrial activity of SCAPs after 1 h, 24 h, and 7 days post-irradiation with grading output powers (0.5, 1, 1.5, and 2 W) for 5 s, in 4 cycles, with resting periods in between, Kruskal–Wallis test, ** *p* < 0.01, *** *p* < 0.001, **** *p* < 0.0001.

One hour after exposure to the 940 nm laser, increased mitochondrial activity was observed when 1 W and 1.5 W output powers were applied (Figure 8). A similar trend continued after 24 h, with no significant difference in the 0.5 W and 2 W groups. Seven days post-irradiation, the mitochondrial activity did not differ significantly from untreated cells.

## 4. Discussion

This study assessed the heat transfer during diode laser irradiation with varying output powers for root canal disinfection in both mature and immature roots. Additionally, structural changes to the inner root canal wall were examined, along with the SCAPs mitochondrial activity when the same irradiation protocols used for endodontic disinfection were applied. The results, in line with biologically accepted temperature limits for external root surface variation [32], suggest that immature roots are more susceptible to overheating during laser disinfection. A previous study reported a nearly three times higher surface temperature of mandibular incisors in comparison to maxillary incisors during high-temperature gutta-percha root canal obturation, suggesting that the remaining radicular dentin affects heat transfer through tissue [33]. The mesial root surface of mandibular incisors, teeth with the lowest dentin volume [34], was used in our study to register temperature values. In this way, the most unfavorable conditions regarding the dentin volume were produced.

After the crown of a tooth is fully formed, the root begins to develop, primarily from dentin. By the time the tooth initially erupts, about half to two-thirds of the root length has already developed, with the remaining development occurring over the next 2–3 years. Following eruption, root development progresses with elongation and an increase in the dentin volume, while the pulpal root space gradually narrows. During this critical stage of root development, when endodontic treatment becomes necessary, treatment options are limited, and the root’s ability to continue developing is compromised [35]. Thus, laser application in RET is investigated on a model of immature teeth [25], so possible overheating during a diode laser application could be investigated. Seraj et al. [36] previously determined the susceptibility of primary teeth to overheating when a 980 nm laser was applied. Therefore, this study aimed to investigate the influence of root development on heat transfer. Linear regression analysis revealed the inverse association between the dentin thickness and temperature values, as thinner root segments corresponded to higher temperature values. Since the only dependent factor was the samples’ dentin volume, our results indicated that the root wall thickness could affect the propagation of heat through tissue, as previously reported in other studies when diode laser irradiation [23], injectable gutta-percha for root obturation [33], and Er:YAG laser irradiation [37] were applied.

The trend of the temperature rising from the cervical to apical third was also observed. In a previous study, the highest temperature values during laser irradiation were registered in the cervical root segment [20]. Contradictory results could be explained by different irradiation protocols. In the mentioned study, the laser fiber was activated during a single 20 s irradiation cycle, moving from the apical to the cervical end and then returning to the apical end. In contrast, the present study followed the previously recommended protocol [23], where fiber helicoidal movement from the apical to cervical end in consecutive cycles, followed by resting periods, was applied. Based on the findings in this study, a 1 W output power could be regarded as safe in immature samples, particularly for the apical third of the root, while 1.5 W can be used for the mature mandibular incisor.

Regarding the influence of the laser parameters, the heating effect was shown to be directly conditioned by a higher output power. Shebab et al. [38] investigated 20 s and 60 s irradiation cycles on mandibular molars with a 1 W, 1.5 W, and 2 W of output power. The study showed that all used powers are safe to apply in mature roots. The different findings could be explained by the different wavelengths of lasers used in the study. It was reported previously that the laser wavelength and output power, the duration of irradiation, as well as the nature of irradiated tissue all contribute to surface heating [39]. Namely, when 810 nm and 980 nm lasers were compared for the temperature rise during the endodontic disinfection protocol, the 810 nm group was found to induce a significantly lower temperature rise on the external root surface when a 1.5 W power was applied in a continuous mode [36]. In an in vitro study on a human canine [20], irradiation with a 980 nm diode laser was reported to be safe both in dry canals or canals filled with distilled water at 1.5 W in a continuous mode. The high dentin volume of human-canine roots in a previous study [20], in comparison to the low dentin volume of roots near the apex [40] and mandibular incisors in the present study, could provide reasons for different recommendations regarding safe laser endodontic application. All mentioned examples suggest that the laser wavelength, as well as the remaining dentin volume, considerably affect the heating effect of diode lasers during endodontic treatment.

The transmission of laser energy through dentin is conditioned by the dentin thickness, tissue color, the degree of sclerosis, and dentinal tubule orientation [41]. The difference in temperature on the inner and outer root walls suggests that some of the laser energy was delivered to dentin, which could explain the structural analysis findings. The overall dentin open porosity on the canal wall was significantly reduced after irradiation, as well as the inner wall dentin density. Lasers of high energy in contact with dentin cause the melting and recrystallization of dentin, while tubule fluid was vaporized in the process [42]. It has quite a useful effect in reducing dentinal hypersensitivity. The ultrastructural examination of the root canal dentin surface revealed a modified amorphous organic matrix, fused intertubular dentin, and the partial opening of the tubules following irradiation with a 970 nm diode laser. In contrast, irradiation with an 808 nm diode laser caused erosion of the intertubular dentin, leading to an irregular surface [13]. In another study, an increase in dentin micro tensile bond strength was observed following irradiation with a 970 nm laser [43]. Also, the 980 nm irradiation with 1.5 W and 3 W settings was found to alter the dentin morphology, but did not affect the root fracture resistance [44].

Photobiostimulation has been extensively studied in the context of low-level laser therapy [45]. However, the effects of high-power irradiation, typically used for root canal disinfection, remain underexplored, particularly regarding the impact of high energy applied to cells over a short period. Divya et al. [12] reported significant periapical healing in the first six months post-treatment, along with notable a bacteria reduction in the 810 nm laser-assisted group. Our findings showed that short exposure to diode laser irradiation at 1 W and 1.5 W significantly stimulated the SCAPs mitochondrial activity within the first 24 h post-irradiation. In contrast, no notable change in mitochondrial activity was observed with the lower power of 0.5 W, nor with the highest power tested (2 W). In the study of Migliraio et al. [46], pre-osteoblasts were irradiated with grading energy outputs (1–50 J). While the peak of cell proliferation occurred at 10 J, higher energy levels reached the limit of cell stimulation. Moreover, a higher level of energy stimulated a dose-dependent generation of oxidative stress that impaired the cells’ physiological functions. These results are in accordance with those obtained in our study, where 2 W (40 J of total radiant energy) did not stimulate cell proliferation 24 h post-irradiation, while lower energy significantly stimulated SCAP proliferation. In the study by Gholami et al. [47], a continuous mode of 0.1 W was applied during short exposures over three days on inflamed periodontal ligament stem cells to explore potential photobiomodulation. Although no significant increase in mitochondrial activity was reported with the low output power, a trend toward increased mitochondrial activity was observed. Nevertheless, the irradiated groups in that study demonstrated a significant rise in the expression of markers for osteogenic differentiation and alkaline phosphatase activity.

Temperature measurements were performed with the thermographic camera on extracted teeth samples in controlled environment conditions. The majority of studies that investigated temperature variations on root surfaces used thermocouples. Each thermocouple measures temperature only on a surface contact point, which could limit data collection. Infrared thermography has many advantages over thermocouples, and it has been widely used in detecting temperature variations in the field of dentistry [48]. Its main advantages represent the capacity to measure the temperature on the wider surface area and the identification of temperature extreme points. The method proved to be a reliable tool for temperature detection on the root surface when the safety of the diode laser application [26] or hot gutta-percha obturation [33] of the root canal is investigated.

The current approaches for investigating a diode laser in endodontics, particularly for its application in regenerative endodontics, face several challenges and limitations. These limitations are primarily related to the complexities of the clinical environment, the properties of the lasers used, and the precision required for ensuring safety and efficacy. There is no universally accepted or standardized protocol for laser application in endodontics, especially when it comes to power settings, exposure times, and safety thresholds for different tooth anatomies. Variations in these parameters can lead to inconsistent results, making it difficult to compare studies or implement clinical guidelines.

## 5. Conclusions

Immature teeth, with thinner dentin walls, were more susceptible to overheating during laser disinfection, especially in the apical third of the root, and a significant rise in temperature was observed for 1, 1.5, and 2 W. A linear regression analysis found an inverse relationship between the dentin thickness and temperature rise, confirming that thinner root segments (with less dentin volume) led to higher temperature increases.

The temperature tended to rise from the cervical to the apical third. Different irradiation protocols could explain some discrepancies, as this study employed a helicoidal fiber movement protocol with resting periods, which may differ from other studies’ protocols.

A 1 W output power was found to be safe for immature teeth, particularly in the apical root third, while 1.5 W could be safely used for mature mandibular incisors.

The significant stimulation of SCAPs mitochondrial activity by diode laser irradiation was found when 1 W and 1.5 W were applied, within the first and 24 h post-irradiation, indicating a potential photobiostimulatory effect. However, no significant changes were observed at lower (0.5 W) and higher (2 W) output powers.

A structural analysis revealed that the dentinal tubule fluid was vaporized during irradiation, leading to the significant melting and recrystallization of dentin. The structural changes to the inner root canal walls indicated an altered dentin microstructure following laser irradiation.

Future studies should explore the impact of irrigants used between irradiation cycles, the potential benefits of conical laser tips for more even energy distribution, and a thorough analysis of how disinfection protocols affect both the dentin structure and stem cell viability.

## Figures and Tables

**Table 1:** The mesial root wall thickness (mean ± SD) in cervical, middle, and apical root third (mm), in instrumented mature and immature root samples.

	Mature Roots (mm)			Immature Roots (mm)		
root third	cervical	middle	apical	cervical	middle	apical
Mean ± SD	1.53 ± 0.17	1.25 ± 0.11	0.58 ± 0.08	1.18 ± 0.22	0.72 ± 0.11	0.34 ± 0.05

**Table 2:** Peak temperature variations at the end of irradiation cycles in all studied groups (°C), in cervical, middle, and apical root third.

Output Power (W)	Root Third	Mature Roots (°C)	Immature Roots (°C)
0.5	cervical	1.67 ± 0.56	1.94 ± 0.17
	middle	1.9 ± 0.44	2.54 ± 0.42
	apical	2.69 ± 1.44	2.86 ± 0.42
1	cervical	2.82 ± 0.46	3.96 ± 0.52
	middle	3.36 ± 0.66	4.84 ± 1.04
	apical	3.6 ± 1.44	5.06 ± 1.09
1.5	cervical	4.49 ± 1.58	6.72 ± 1.19
	middle	5.94 ± 1.55	8.70 ± 2.27
	apical	7.21 ± 1.64	10.43 ± 1.60
2	cervical	5.48 ± 0.86	9.48 ± 0.99
	middle	7.52 ± 1.76	10.96 ± 1.41
	apical	8.11 ± 1.67	12.08 ± 0.7

**Table 3:** Calculated temperature variations at the end of irradiation cycles in all studied groups (°C), in cervical, middle, and apical root third on inner mesial walls.

Output Power (W)	Root Third	Mature Roots (°C)	Immature Roots (°C)
0.5	cervical	1.97	3.24
	middle	3.2	3.94
	apical	3.99	4.16
1	cervical	5.42	6.56
	middle	5.95	7.44
	apical	6.2	7.66
1.5	cervical	8.39	10.62
	middle	9.84	12.6
	apical	11.11	14.3
2	cervical	10.68	14.68
	middle	12.72	16.6
	apical	13.31	17.26

## Data Availability

The original contributions presented in this study are included in this article/Supplementary Materials. Further inquiries can be directed to the corresponding author.

## Supplementary Materials

The following supporting information can be downloaded at: https://www.mdpi.com/article/10.3390/biomimetics10040216/s1, Figure S1: A—diode laser, thermography camera and tooth sample positioned for recording, B—laser setting display, C—thermography camera during irradiation recording temperature in root segments; Figure S2: Representative figures of micro-CT scanning of samples, A—2D reconstruction of tooth, B—3D tooth model reconstructed, C—region of interest as zone of demineralization.; Table S1: Laser irradiation parameters; Table S2. Absolute values for temperature recorder on external mesial root wall during irradiation with 940 nm laser with output power of 1.5 W, in 4 consecutive cycles.

## Appendix A

### Appendix A.1. Calculations of Temperature on the Inner Dentinal Walls

A brief theoretical explanation of the calculation of the increase in temperature on the inner surface of dentin during various laser treatment.

Starting from the assumption that that tooth possesses a typical composition structure, where from one side, dentin and enamel and the other, dentin and cementum form specific mutually interconnected layers, it is possible from the thermal conductivity of particular layers to determine the apparent thermal conductivity λ_app_ of both layers, using the following equation:

(A1) $$\frac{1}{\lambda_{app}}=\left(\frac{H_{cementum}/\lambda_{cementum+{H_{dentine/}}_{\lambda_{dentine}}}}{H_{cementum+}H_{dentine}}\right),$$

where (H_cementum_, H_dentin_) and (λ_cementum_, λ_dentin_) are the thickness and effective thermal conductivity of the cementum and dentin layers, respectively.

Assuming that H_cementum_ in the root apex is 0.15 mm and dentin is 0.97 mm, and the thermal conductivity of cementum is 0.628 W/mK and of dentin 0.57 W/mK, the calculated value of the effective thermal conductivity of both layers λ_app_ is 0.58 W/mK.

Knowing that the coefficient of the heat transfer K can be expressed by:

K_app_ = λ_app_/H, (A2)

where H = H_cementum_ + H_dentin_, the calculated K_app_ is 5.2 × 10^2^ W/m^2^K.

Firstly, we can assume that the geometry of the inner surface of the teeth within the heat transfer by the conduction which occurred can be presented by a truncated cone. The base of this cone then is a smaller circle, for which the radius is r, while the top surface is the larger circle, with a radius of R. The slant height is the shortest distance between the two circles, while the lateral surface is the surface without the circles, which can be calculated using the following equation:

(A3) L=(R+r)πs,

where $=\sqrt{{\left(R-r\right)}^{2}+h^{2}}$.

Assuming that R = 0.2 mm and r = 0.35 mm, then the calculated value of L = 174.7 mm^2^ can be taken from them, while the total surface is S = L + R^2^π. Since the surface of the base is 12.6 mm^2^, it follows that the total area of the inner surface of the truncated cone is 187.3 mm^2^.

The area of the outer surface can be obtained in a similar way, by the addition of the total thickness of the dentin and cementum to the inner radius of the void top and base circle. In this case, the lateral surface is 251.2 mm^2^ and total surface 281.8 mm^2^, while the average value of the total surface is 219.2 mm^2^.

Now, following this expression, which describes the transferred apparent heat through the surface area S, in time t,

(A4) $$Q=\frac{\lambda_{app}}{H}(T_{1}-T_{2})\mathrm{St}=K_{\mathrm{app}}\;(T_{1}-T_{2})St$$

where hat T_2_ is temperature in the outer area of cementum and T_1_ is the temperature in the inner area of dentin, S is the average area through which heat is transferred, H is the total thickness of dentin and cementum in the apical area, assuming that the value of transmittance is 70%, as shown in the ref., and then the transferred laser energy in heat in the cycle is Q = 0.3E, while:

(A5) $$\Delta T=T_{1}-T_{2}=0.3E/K_{app}St$$

Taking into account the values for a laser power of 0.5 W, or energy E = 2.5 J emitted in the first cycle, if we take previously calculated values of the apparent heat transfer K_app_ = 5.2 × 10^2^ W/m^2^K and the average surface of the truncated cone S = 219.2 mm^2^, where a thorough heat transfer has occurred, then we can calculate the temperature difference between the outer surface (cementum surface) and inner surface (dentin surface). In a state of the heat equilibrium inside the total truncated cone, it follows that ΔT_11_ − ΔT_12_ = 1.3 °C, where ΔT_11_ is the temperature of the inner dentin surface, while ΔT_12_ is the temperature of the outer cementum surface.

Taking into account that after 5 s of laser treatment with a power of 0.5 W, the measured change of the temperature of the outer cementum surface of the cervical root third was ΔT_12_ = 1.67 °C, the increase in the temperature of the inner dentin surface of mature roots ΔT_11_ should be 1.3 + 1.67 = 2.97 °C. For dentin in the middle root third of mature roots, the calculated value is 3.2 °C, and dentin on the apical root third of mature roots has a value of 3.99 °C. This calculation assumed that a time of 20 s between various stages of laser heating is enough to decrease the temperature of the root walls on the level of the previous stage. For immature roots, dentin temperatures obtained in the same way for the cervical, middle, and apical rooth third were 3.24 °C, 3.94 °C, and 4.16 °C, respectively.

For a laser power of 1 W, the calculated value for ΔT = ΔT_21_ − ΔT_22_ = 2.6 °C. The calculated values of the ΔT_21_ inner-surface dentin for the cervical, middle, and apical root third for mature roots are 5.42 °C, 5.95 °C, and 6.2 °C, while for immature roots, they are 6.56 °C, 7.44 °C, and 7.66 °C, respectively.

For a laser power of 1.5 W, the calculated value for ΔT = ΔT_31_ − ΔT_32_ = 3.9 °C. The calculated values of the ΔT_31_ inner-surface dentin for the cervical, middle, and apical root third for mature roots are 8.39 °C, 9.84 °C, and 11.11 °C, while for immature roots, they are 10.62 °C, 12.6 °C, and 14.33 °C, respectively.

For a laser power of 2 W, the calculated value for ΔT = ΔT_41_ − ΔT_42_ = 5.2 °C. The calculated values of the ΔT_31_ inner-surface dentin for the cervical, middle, and apical root third for mature roots are 10.68 °C, 12.72 °C, and 13.31 °C, while for immature roots, they are 14.68 °C, 16.6 °C, and 17.26 °C, respectively.

### Appendix A.2. Calculation of Laser Irradiation Parameters

#### Appendix A.2.1. Power Density/Irradiance at Target (mW/cm^2^)

The power density or irradiance refers to the amount of a laser power incident on a unit area of the target. It is usually measured in mW/cm^2^. The formula for the power density *I* is given by:

$$I=\frac{P}{A}$$

where *P* is the laser power in mW (milliwatts) and *A* is the area of the target in cm^2^ (square centimeters).

The calculation for 0.5 W for root irradiation is as follows: *I*_(0.5W)_ = 500 mW/1.873 cm^2^ = 267 mW/cm^2^.

#### Appendix A.2.2. Radiant Energy (J)

Radiant energy refers to the total energy emitted by the laser in the form of light, usually measured in joules (J). The formula for radiant energy *E* is given by:

E=P×t

where *P* is the laser power in W (watts, where 1 W = 1 J/s) and *t* is the time the laser is active in seconds (s). The calculation for 0.5 W for root irradiation is as follows: *E*_(0.5W)_ = 0.5 W × 5 s = 2.5 J.

#### Appendix A.2.3. Energy Density (J/cm^2^)

Energy density refers to the amount of radiant energy delivered per unit area, expressed in J/cm^2^.

The formula for energy density *H* is:

$$H=\frac{E}{A}$$

where *E* is the radiant energy in J and *A* is the area in cm^2^ over which the energy is distributed. The calculation for 0.5 W for root irradiation is as follows: *H*_(0.5W)_ = 2.5 J/1.873 cm^2^ = 1.33 J/cm^2^.

#### Appendix A.2.4. Total Radiant Energy (J)

Since the laser operated for four cycles, the total radiant energy for 0.5 W for root irradiation is:

*E*_(4cycles, 0.5W)_ = 2.5 J × 4 = 10 J

All calculated parameters are represented in Table A1.

**Table A1:** Laser irradiation parameters.

Parameter	Teeth Sample Irradiation	SCAPs Irradiation
Laser type	Diode laser (InGaAsP)	Diode laser (InGaAsP)
Wavelength (nm)	940 ± 10	940 ± 10
Operating mode	Continuous wave	Continuous wave
Average radiant power (mW)	500, 1000, 1500, 2000	500, 1000, 1500, 2000
Beam spot size/area at target (cm^2^)	1.873	0.33
Distance from target (mm)	/	13
Exposure duration (s)	5	5
Power density/irradiance at target (mW/cm^2^)	267, 534, 801, 1068	1515, 3030, 4545, 6060
Radiant energy (J)	2.5, 5, 7.5, 10	2.5, 5, 7.5, 10
Energy density/radiant exposure (J/cm^2^)	1.33, 2.67, 4, 5.34	7.6, 15, 22.7, 30.3
Number and frequency of treatment sessions	4 cycles, with 20 s resting periods	4 cycles, with 20 s resting periods
Total radiant energy (J)	10, 20, 30, 40	10, 20, 30, 40
