# Supplementary material for: A Biomimetic Approach to Diode Laser Use in Endodontic Treatment of Immature Teeth: Thermal, Structural, and Biological Analysis
Source: Biomimetics (Basel). 2025 Apr 2;10(4):216. doi: 10.3390/biomimetics10040216 (PMC12025333; doi:10.3390/biomimetics10040216)
Supplement: Supplementary file 1 [file biomimetics-10-00216-s001.zip › biomimetics-3488251-supplementary.pdf]

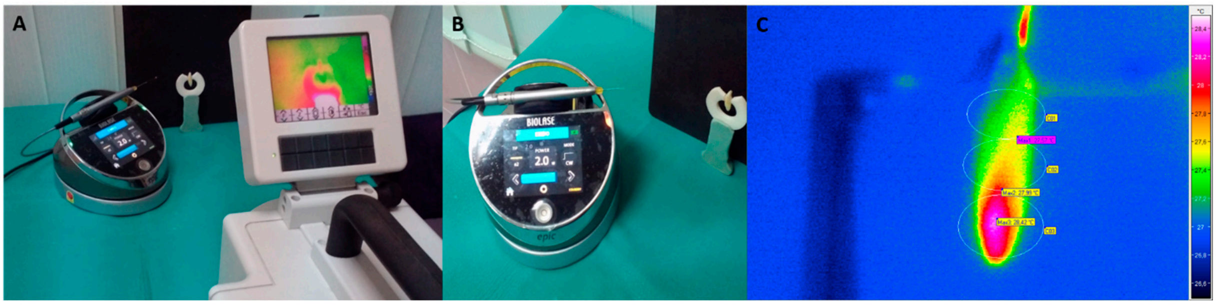

Figure S1. A – diode laser, thermography camera and tooth sample positioned for recording, B – laser setting displey, C – thermography camera during irradiation recording temperature in root segments

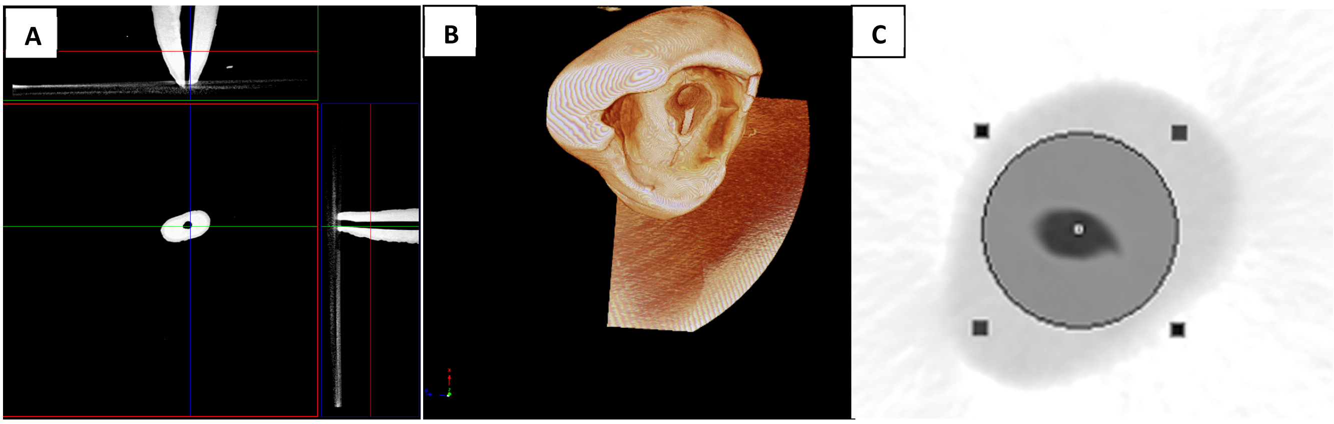

Figure S2. Representative figures of micro-CT scanning of samples, A – 2D reconstruction of tooth, B – 3D tooth model reconstructed, C - region of interest as zone of demineralization.

Table S1. Laser irradiation parameters

Parameter	Teeth Sample Irradiation	SCAPs Irradiation
Laser type	Diode laser (InGaAsP)	Diode laser (InGaAsP)
Wavelength (nm)	940 ± 10	940 ± 10
Operating mode	Continuous wave	Continuous wave
Average radiant power (mW)	500, 1000, 1500, 2000	500, 1000, 1500, 2000
Beam spot size/area at target (cm^2)	1.873	0.33
Distance from target (mm)	/	13
Exposure duration (s)	5	5
Power density/irradiance at target (mW/cm^2)	267, 534, 801, 1 068	1 515, 3 030, 4 545, 6 060
Radiant energy (J)	2.5, 5, 7.5, 10	2.5, 5, 7.5, 10
Energy density/radiant exposure (J/cm^2)	1.33, 2.67, 4, 5.34	7.6, 15, 22.7, 30.3
Number and frequency of treatment sessions	4 cycles, with 20 s resting periods	4 cycles, with 20 s resting periods
Total radiant energy (J)	10, 20, 30, 40	10, 20, 30, 40

Table S2. Absolute values for temperature recorder on external mesial root wall during irradiation with 940 nm laser with output power of 1.5 W, in 4 consecutive cycles.

Time (s)	cervical	middle	apical
0	21.848	21.94	21.97
1.47	22.148	22.362	22.507
5.42	23.331	23.777	24.265
7.66	25.605	26.092	26.849
9.66	25.255	25.892	26.549
17.68	24.487	25.014	25.554
20.65	24.386	24.82	25.3
21.67	24.291	24.76	25.203
23.69	24.27	24.725	25.1
24.66	24.254	24.699	25
26.62	24.23	24.668	24.9
27.63	24.22	24.556	24.854
32.42	25.887	26.37	26.974
33.5	26.235	27.017	27.7
35.52	25.871	26.593	27.32
37.54	25.756	26.293	26.993
42.8	25.398	25.74	26.362
47.26	25.226	25.487	25.996
53.63	25.037	25.315	25.635
56.87	25.76	26.119	26.909
59.89	26.84	27.869	28.919
64.91	26.199	26.99	27.582
67.94	25.998	26.65	27.118
70.96	25.779	26.3	26.72
73.27	25.694	26.1	26.512
76.71	25.584	25.87	26.199
79.97	25.497	25.85	26.179
85.99	27.597	28.967	29.934
90.07	26.988	28.066	28.867
95.14	26.462	27.241	27.747
100.22	26.246	26.934	27.223
105.3	26.16	26.762	26.903
